# Validity and Reliability of a Telehealth Physical Fitness and Functional Assessment Battery for Ambulatory Youth With and Without Mobility Disabilities: Observational Measurement Study

**DOI:** 10.2196/50582

**Published:** 2024-02-12

**Authors:** Byron Lai, Danielle Wadsworth, Katherine Spring, Chloe S Jones, Madison Mintz, Laurie A Malone, Yumi Kim, Jereme Wilroy, Holim Lee

**Affiliations:** 1 Division of Pediatric Rehabilitation Medicine University of Alabama at Birmingham Birmingham, AL United States; 2 Exercise Adherence and Obesity Prevention Laboratory School of Kinesiology Auburn University Auburn, AL United States; 3 Pennington Biomedical Research Center Division of Population and Public Health Science Pediatric Obesity and Health Behavior Laboratory Baton Rouge, LA United States; 4 Department of Occupational Therapy School of Health Professions University of Alabama at Birmingham Birmingham, AL United States; 5 Department of Physical Medicine & Rehabilitation University of Alabama at Birmingham Birmingham, AL United States

**Keywords:** cerebral palsy, telehealth, young adults, telemonitoring, exercise, therapy, therapeutic exercise, assessment, teleassessment, reliability, usability, disability, youth, physical fitness, videoconference

## Abstract

**Background:**

Youth (age 15-24 years) with and without disability are not adequately represented enough in exercise research due to a lack of time and transportation. These barriers can be overcome by including accessible web-based assessments that eliminate the need for on-site visitations. There is no simple, low-cost, and psychometrically sound compilation of measures for physical fitness and function that can be applied to youth with and without mobility disabilities.

**Objective:**

The first purpose was to determine the statistical level of agreement of 4 web-modified clinical assessments with how they are typically conducted in person at a laboratory (convergent validity). The second purpose was to determine the level of agreement between a novice and an expert rater (interrater reliability). The third purpose was to explore the feasibility of implementing the assessments via 2 metrics: safety and duration.

**Methods:**

The study enrolled 19 ambulatory youth: 9 (47%) with cerebral palsy with various mobility disabilities from a children’s hospital and 10 (53%) without disabilities from a university student population. Participants performed a battery of tests via videoconferencing and in person. The test condition (teleassessment and in person) order was randomized. The battery consisted of the hand grip strength test with a dynamometer, the five times sit-to-stand test (FTST), the timed up-and-go (TUG) test, and the 6-minute walk test (6MWT) either around a standard circular track (in person) or around a smaller home-modified track (teleassessment version, home-modified 6-minute walk test [HM6MWT]). Statistical analyses included descriptive data, intraclass correlation coefficients (ICCs), and Bland-Altman plots.

**Results:**

The mean time to complete the in-person assessment was 16.9 (SD 4.8) minutes and the teleassessment was 21.1 (SD 5.9) minutes. No falls, injuries, or adverse events occurred. Excellent convergent validity was shown for telemeasured hand grip strength (right ICC=0.96, left ICC=0.98, *P*<.001) and the TUG test (ICC=0.92, *P*=.01). The FTST demonstrated good agreement (ICC=0.95, 95% CI 0.79-0.98; *P*=.01). The HM6MWT demonstrated poor absolute agreement with the 6MWT. However, further exploratory analysis revealed a strong positive correlation between the tests (r=0.83, *P*<.001). The interrater reliability was excellent for all tests (all ICCs>0.9, *P*<.05).

**Conclusions:**

This study suggests that videoconference assessments are convenient and useful measures of fitness and function among youth with and without disabilities. This paper presents operationalized teleassessment procedures that can be replicated by health professionals to produce valid and reliable measurements. This study is a first step toward developing teleassessments that can bypass the need for on-site data collection visitations for this age group. Further research is needed to identify psychometrically sound teleassessment procedures, particularly for measures of cardiorespiratory endurance or walking ability.

## Introduction

In clinical trials of exercise, conventional measures of physical fitness and function (2 determinants of successful interventions) require participants to be physically present at a laboratory to undergo measurement procedures with specialized equipment. This requirement is burdensome and time-consuming, which negatively affects enrollment rates. In fact, 2 of the most common reasons for nonparticipation in exercise interventions are a lack of time and transportation [1,2]. To overcome these challenges, exercise trials have begun to use web-based videoconferencing to implement intervention protocols and, more recently, collect study outcome data (ie, teleassessments). The obvious benefit of teleassessments is that they negate the need for participants to travel to an on-site research facility. This benefit is critical for advancing scientific knowledge in exercise research.

Clinical exercise interventions are limited by their ability to reach a representative sample size, and this limits the generalizability of study findings. Systematic reviews of exercise research among young adults have reported that clinical trials lacked representativeness. One review reported that only 77% of studies achieved their recruitment targets [3]. Another review reported that 86% of adults who participated in exercise research were Caucasian (mean age 51 years) [4]. Representativeness was worse among clinical populations. Considering people with physical disabilities, reviews have found that the average sample size for randomized controlled trials of exercise is 30 people: 15 per treatment and 15 per control group [1,5,6]. Moreover, a review found that 58.9% of adults with physical disabilities who were contacted to participate in exercise trials were lost before study enrollment and an even smaller percentage of people completed the exercise trial or returned for their follow-up data collection [1]. There is a genuine need for accessible and inclusive ways to increase participation in exercise trials, given that 1 in 4 adults in general and 1 in 2 adults with physical disabilities in the United States do not meet the national guidelines for exercise [7-10]. Achieving the national guidelines for exercise is important for preventing and managing all-cause morbidity and mortality [5,7,11,12].

There are existing studies on telehealth assessments, particularly among middle-aged and older adults [13-20]. Relevant prior works included a study that investigated mobility-focused physical outcome measures, which included the hand grip strength test, the five times sit-to-stand test (FTST), and the timed up-and-go (TUG) test [19]; multiple studies have investigated a remotely delivered version of a 6-minute walk test (6MWT) [21-23]; and a pilot investigated balance and gait assessments [24]. The 6MWT has also been found to be a valid indicator of cardiorespiratory fitness [25-27]. There were similar teleassessment investigations with the movement assessment battery for children (5-11 years old) [28], as well as the TUG test in children and teenagers (6-18 years old) with autism spectrum disorder [29]. Notably, a systematic review found that teleassessments had strong psychometric properties among adults [20], but there are far less investigations among younger age groups, particularly younger age groups with difficulties in gross motor function.

The youth demographic, defined as persons aged from 15 to 24 years according to the United Nations and the World Health Organization (WHO), is important because this is the age range where people adopt sedentary lifestyles that last throughout adulthood. There are 3 reasons why exercise promotion is important among youth: (1) data demonstrate that exercise participation levels are alarmingly low and continue to decline throughout the youth age range [30-33], particularly among youth with disabilities [34-36]; (2) adoption of exercise behavior during youth may increase the likelihood that people are regular exercisers in adulthood [37,38]; and (3) exercise during youth may prevent obesity and cardiometabolic disease in adulthood [39,40]. Moreover, the youth age range is where clinical populations tend to experience functional decline [41]. One study found that people with cerebral palsy (CP) with mobility disabilities experience clinically significant declines in physical function as they age from adolescence to adulthood [42]. Another study on youth with CP found that the probability of walking is highest at age 9 years (68%) and lower at age 18 years (approx. 50%) [43]. Two other studies have revealed the same pattern of functional loss and called for a more comprehensive therapeutic approach beyond the traditional focus on childhood [44,45].

Making an impact on exercise participation will require telehealth-driven exercise trials, with teleassessments that are inclusive of youth with and without disabilities. Inclusive trials are important not only for health promotion but also for disability equity, as fundamentally described in the *First Global Physical Activity and Sedentary Behavior Guidelines for People Living with Disability*, released by WHO: “Creating opportunities for inclusion in physical activity for people living with disability can help eliminate such barriers by changing perceptions, emphasizing strengths and abilities, promoting personal resilience, and having an onward impact on inclusion in society” [46,47].

Teleassessments that support large-scale exercise trials should include safe, valid, and reliable methods with affordable equipment. Nevertheless, there are few established methods among the youth age range. Additionally, there has not been a psychometric evaluation of a standardized compilation of teleassessments (ie, teleassessment battery) that is inclusive of youth with and without disabilities. Thus, this study investigated the validity and reliability of an inclusive teleassessment battery. The battery included physical tests for indicators of physical fitness and function that could be performed at home through videoconferencing.

Specifically, our study had the following 3 purposes:

- Primary purpose: to determine the level of convergent validity between tele- and in-person assessments of exercise among youth with and without disabilities by comparing the agreement between test results using the intraclass correlation coefficient (ICC) for validity (ICC-v). We hypothesized that the teleassessment tests would at least demonstrate good agreement with conventional in-person assessments.
- Secondary purpose: to determine the level of interrater reliability for the teleassessment tests between 2 independent raters using the intraclass correlation coefficient for reliability (ICC-r), specifically among youth with disabilities. We hypothesized that 2 raters would achieve at least good agreement on each rater-dependent test included in the battery.
- Tertiary purpose: to describe the feasibility of the teleassessments through several metrics, such as assessment duration, technical usability issues, perceived barriers and facilitators with the setup and main procedure, and potential home implementation.

## Methods

### Design and Overview

This was an observational measurement study evaluating the validity and reliability of a teleassessment battery. The study compared modified teleassessment procedures to the gold standard: in-person evaluations among youth with CP and without disabilities. Data were collected from August 2022 to February 2023. The study aimed to recruit 19 youth, 9 (47%) with CP and 10 (53%) without disabilities. Participants performed 4 physical tests under the following 2 conditions: in person with supervision and videoconference assessments in a simulated home environment. The order in which the assessments were completed was randomized. Both assessments were completed in a single visit to the laboratory.

### Recruitment Criteria and Process

The general eligibility criteria were as follows: (1) age 15-24 years and (2) the ability to understand instructions and communicate in English. Additional eligibility criteria for people with disabilities included (1) self-reported mobility disability and (2) ability to walk 20 feet with or without assistance from a caregiver or mobility device. The presence of any orthopedic, vascular, cardiac, or other health-related issue that could make the study procedures unsafe was considered an exclusionary criterion.

People with disabilities were recruited from the medical and billing record databases of the Children’s Hospital from the Division of Pediatric Rehabilitation Medicine, which works with a diverse group of children and youth with disabilities. People without disabilities were recruited from the student population of Auburn University and were age- and sex-matched to participants with disabilities. Recruitment strategies included referrals, study flyers, mailouts, and word of mouth.

### Power Analysis and Sample Size Justification

This study aimed to enroll a sample size of 19 individuals to satisfy an ICC power calculation with the following components for the primary study purpose: statistical power (1 – β)=0.8; α=.05; 2 observations; H_0_=0.7, H_1_=0.9 [48].

### Measures

A total of 4 tests were included to assess physical fitness and motor function. Tests that require complex coordination or precise timing were not considered due to feasibility concerns. The tests were chosen based on their feasibility and safety to be performed in an average home setting [49], their broad use in research and clinical settings, and their well-researched psychometric properties in the adult population with and without disabilities [50-59]. The teleassessment protocols were modified to better suit the home environment. Picture demonstrations and instructions are included in Multimedia Appendix 1.

The tests were conducted in the following order: the hand grip strength test with a dynamometer, the FTST, the TUG test, and the 6MWT.

#### Hand Grip Strength Test (Physical Fitness)

The participants were instructed to sit in a stationary chair using a Camry digital hand dynamometer. The procedure included 3 trials with each hand, with the elbow flexed at 90°, with a 30-second rest in between trials. For videoconference assessments, the field of view included the participant’s upper body. The participants were instructed to position the laptop camera to include their elbow, the device, and their face to ensure the posture was correct. Several studies have supported the validity and reliability of this test among a variety of populations [51-55].

#### Five Times Sit-to-Stand Test (Physical Fitness)

**Figure 1:**
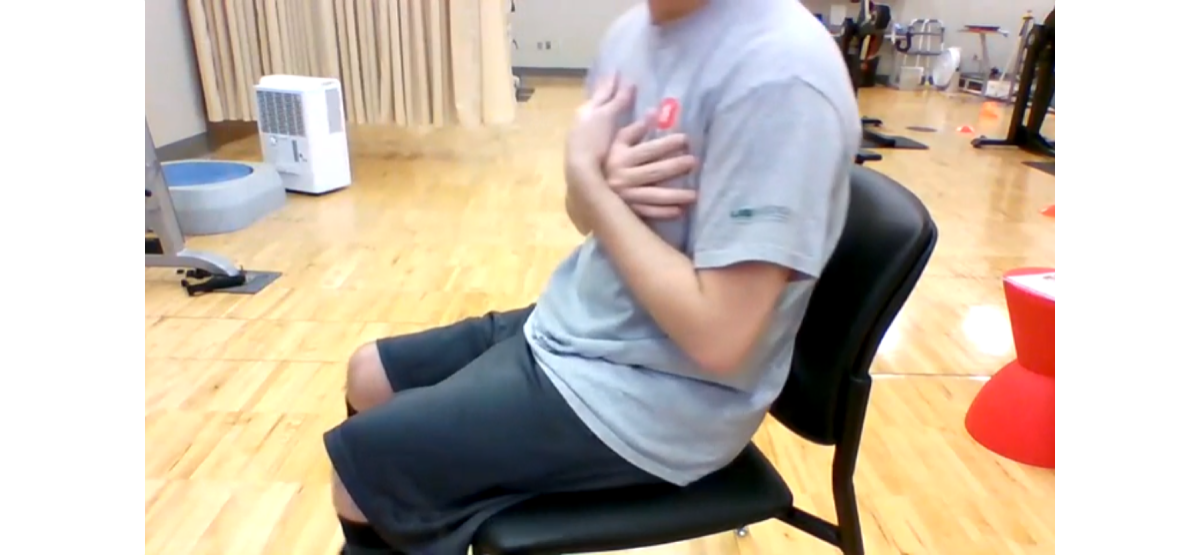
Laptop camera view of the FTST. FTST: five times sit-to-stand test.

The equipment included a chair, 24 inches in height, without arm rests. The participants were instructed to sit in the chair and then stand up and sit down 5 times as fast as they could. The time it took to complete the task was recorded in seconds. For the videoconference assessment, each participant was instructed to rotate the chair 90° so that the recording included a profile view of the participant’s entire body (at least the shoulders, hips, and knees); see Figure 1. A repetition was counted as complete only when the participant’s rear contacted the chair. Several studies have supported the validity and reliability of the FTST [60-63].

#### Timed Up-and-Go Test (Lower Extremity Function)

**Figure 2:**
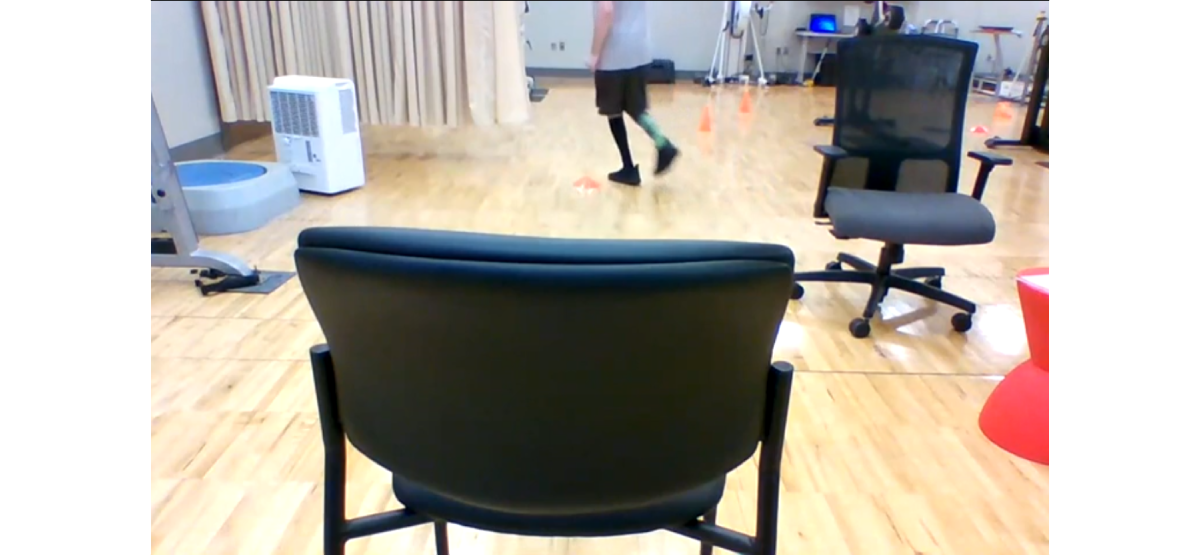
Laptop camera view of the TUG test: TUG: timed up-and-go.

The participants were instructed sit in a chair and then to stand up, walk straight to a cone that was placed 118 inches (3 m) away from the chair, turn around, and walk back to sit down in the chair. The time it took to complete the task was recorded in seconds. For the videoconference assessment, the participants were instructed to rotate the chair 90°. They were then instructed to place down the measuring tape starting from the chair. The tape needed to be straight, without wrinkles or folds. The participants were instructed to adjust the camera angle to include their entire body throughout the test, the floor, the chair, and the entire 3 m walkway (Figure 2). The task was considered complete only when the participant’s rear contacted the chair. The reliability and validity of the TUG test have been demonstrated in a variety of populations [56].

#### Six-Minute Walk Test (Lower Extremity Function and Cardiorespiratory Fitness)

For the in-person 6MWT, participants were instructed to walk as much as possible in 6 minutes around a circular track that was marked by cones. The distance walked was measured with a distance-measuring wheel, which was held by a research staff member, who followed the participant around the track during the test. The 6MWT has a variety of studies supporting its psychometric properties for measuring lower extremity function or walking ability and cardiorespiratory fitness among a variety of populations [21-23,25-27,50,57].

**Figure 3:**
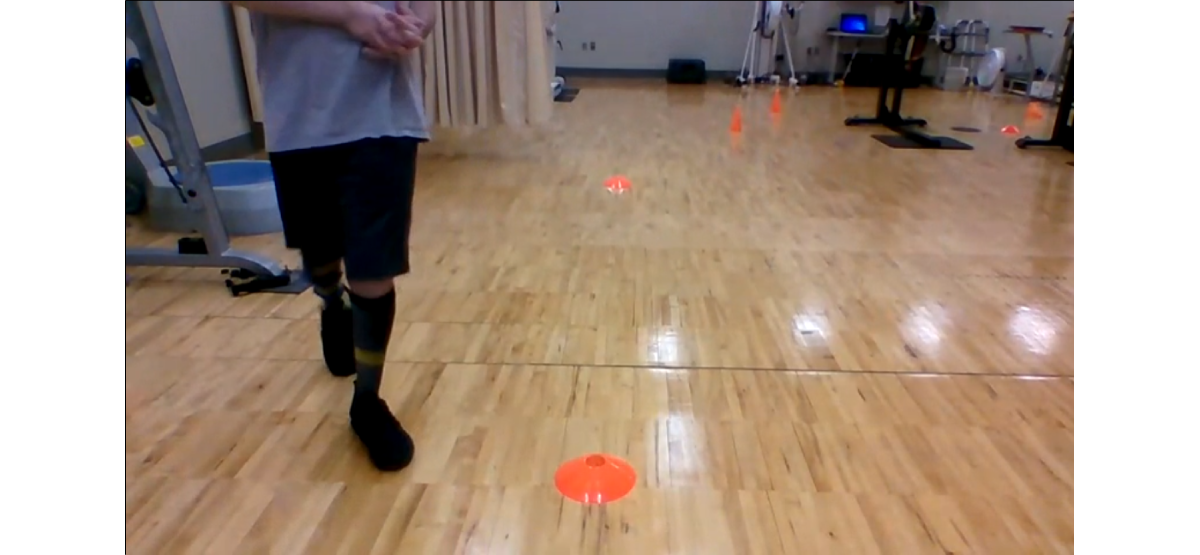
Laptop camera view of the 6MWT: 6MWT: 6-minute walk test.

The research team devised a shorter, home-modified version of the 6MWT to reflect the space constraints often found in a participant’s home (Figure 3). The home-modified 6-minute walk test (HM6MWT) followed the TUG test. Thus, from the previous TUG teleassessment setup, participants were instructed to place an additional cone directly at their feet while sitting in the chair. The participants were then asked to move the chair out of the way of the 2-cone obstacle course. The camera was positioned to include the participant’s entire body throughout the test, the floor, and the entire walkway. The equipment in total included 2 cones and a piece of measuring tape to measure out the 118-inch (3 m) walkway. The assessor counted the number of laps that were completed in 6 minutes. Assessors also estimated the length of the last incomplete lap as a fraction (eg, 0.25 laps) during the 6 minutes.

### Procedures

All participants completed the 2 types of assessments (tele- and in-person assessments) in a single visit. The order in which a participant completed the tele- and in-person assessments was randomized and counterbalanced. In-person assessments were conducted in a typical laboratory setting. Teleassessments were conducted in a different setting; the space for teleassessments was measured to be a minimum of 10 × 15 square feet to resemble a modest estimate of an average living room. The in-person assessments were performed under the supervision of a research staff member, while the videoconference assessments were conducted using Zoom videoconferencing. For the latter, participants set up each teleassessment with the verbal guidance of the research staff member on Zoom. A caregiver was allowed to assist their child in the teleassessment setup and in performing the tests in order to prevent falls that might occur.

The general procedure was as follows: participants were briefed and provided informed consent; they completed the study surveys (demographic information and videoconference literacy), underwent randomization via a coin flip, and completed the tests under both conditions; and then they completed a follow-up questionnaire on their experience with the teleassessments. Videoconference literacy was assessed via the Video Conference Literacy and Usability Questionnaire, which was modified from the Telehealth Usability Questionnaire [64]. The follow-up questionnaire included 3 open-ended questions: (1) likes about the assessments, (2) dislikes about the assessments, and (3) technical issues or problems they experienced during the assessments. Study staff were also instructed to record problems or issues they observed during the assessments on the data collection form.

Regarding the setting, participant groups (youth with and without disabilities) completed the testing at 2 different university laboratories. The protocols for conducting the assessments were matched between the research teams. To assist with the standardization, assessors were given scripts on how to guide participants in setting up the teleassessments and performing each test.

For study purpose 1, 1 research staff member scored all assessments for youth with disabilities (author BL, a disability exercise specialist with over 10 years of clinical experience). Graduate research assistants scored all assessments for youth without disabilities. For study purpose 2, the videoconference recordings of the functional tests part were scored independently by 2 raters (author LM, a senior disability exercise specialist, and a doctoral student in rehabilitation science), who were blinded to the randomization, assessment type, order, and participant and researcher conversations before and after the assessments. The raters were trained to score by the lead investigator (BL) using an operations manual included in Multimedia Appendix 1. Training included a preliminary assessment of interrater reliability for a sample of 3 participants, from which they had excellent agreement for all assessments (>99% absolute agreement for the hand grip strength test, the FTST, and the TUG test; 96% for the 6MWT). The plan was to retrain them if they achieved less than 95% agreement on the assessments. Study purpose 3, feasibility, included several descriptive metrics: the participant feedback survey; duration to complete the assessments in minutes; problems, issues, or nuances experienced during the testing; and observational feedback from the assessors (recorded on the data collection form).

### Equipment

Teleassessment rooms were equipped with a Chromebook brand laptop (Samsung Galaxy Chromebook Professional Laptop, 13.3 inches, with a built-in microphone and web camera). At the start of the teleassessment, the laptop was positioned on the table. Assessment equipment included a hand grip strength dynamometer (CAMRY digital hand grip dynamometer), disc cones, a distance-measuring wheel, and a soft measuring tape that was cut to a 118-inch (3 m) length.

### Analysis

For study purpose 1, ICCs were used to examine the convergent validity (ICC-v) between the test conditions. ICC-v values were complemented with Bland-Altman plots to visualize differences in agreement [65]. For the HM6MWT, additional exploratory analyses were performed to identify the optimal multiplier for the laps that would best estimate the distance in meters obtained from an in-person 6MWT. Specifically, the number of laps was first multiplied by a value of 6 m (cones were laid out 3 m away from each other—hence a minimum track of 6 m) and tested, then multiplied by 7 m, 8 m, and so on until the multipliers for the highest ICC-v were identified. For only the 6MWT, Pearson correlation analysis was planned if agreement analyses were not identified through the ICC-v.

For study purpose 2, ICCs were used to examine the interrater reliability (ICC-r) between 2 assessors (a doctoral student in rehabilitation science and a senior exercise physiology researcher). The assessors scored recorded videos of the teleassessments from the 9 (47%) ambulatory youth with CP, since the study team anticipated higher variability of performance due to mobility disability.

ICCs and their 95% CIs were calculated using IBM SPSS version 24. For the ICC-v, a 2-way mixed-effects model with absolute agreement was used with single or average measures, as appropriate for each test. For the ICC-r, a 2-way random-effects model was used with absolute agreement and single measures. The ICC interpretation criteria were as follows: 0-0.5 was considered poor; 0.5-0.75, moderate; 0.75-0.9, good; and 0.9 or higher, excellent [66]. The ICC analyses were first calculated against ICC H_0_=0.75 to derive the conclusion that the validity or reliability was at least good in terms of agreement, in accordance with the study hypotheses. Further comparison against excellent agreement (ICC H_0_=0.9) was conducted if preliminary analysis identified good agreement.

For study purpose 3 (feasibility), data on the following items were collected: the duration of both types of assessments, technical usability issues, and problems or adverse events experienced by participants or assessors.

### Ethical Considerations

Written informed consent was obtained from all participants prior to their engagement in the study. For completing the study, participants without disabilities were compensated with extra course credit, while participants with disabilities were compensated with a US $60 gift card. The study procedures were conducted separately at each university and approved by the Institutional Review Board of each university (University of Alabama at Birmingham: #300009041; Auburn University: #22-112 EP 2204), with the agreement that study results would be combined for analysis. Participation was kept confidential.

## Results

### Participant Information

Participant characteristics are shown in Table 1. All 9 (47%) youth with mobility disabilities were ambulatory with a primary diagnosis of CP with a Gross Motor Function Classification System Level of I-III; of them, 8 (89%) were described as hemiplegic in terms of motor disability. One required physical assistance from a caregiver while walking, and another wore a right-leg orthotic device during the tests. One person with CP had mild-to-moderate cognitive disability. There were no statistically significant differences between groups in age, height, weight, or other aspects. Participants generally reported high videoconference literacy and usability scores.

**Table 1:** Overall participant characteristics (N=19).

Characteristics		Youth with CP (n=9)	Youth without disabilities (n=10)
**Demographics**			
	Age (years), mean (SD)	17.4 (1.9)	19.3 (1.2)
	Sex (male/female), n (%)	5 (56) male, 4 (44) female	5 (50) male, 5 (50) female
	Height (cm), mean (SD)	160.1 (15)	160 (35)
	Weight (lb), mean (SD)	142.7 (38)	149.6 (29)
**Videoconference literacy and usability questionnaire, mean (SD)**			
	Usefulness	13.2 (1.6)	12.1 (1.9)
	Ease of use and learnability	12.7 (1.9)	13.1 (1.9)
	Interface quality	17.2 (2.6)	15.3 (2)
	Interaction quality	14.1 (2.9)	10.5 (6.8)
	Reliability	10.6 (2.4)	8.8 (2.1)
	Satisfaction and future use	18.6 (1.9)	16.1 (2.7)

### Convergent Validity (Purpose 1)

**Figure 4:**
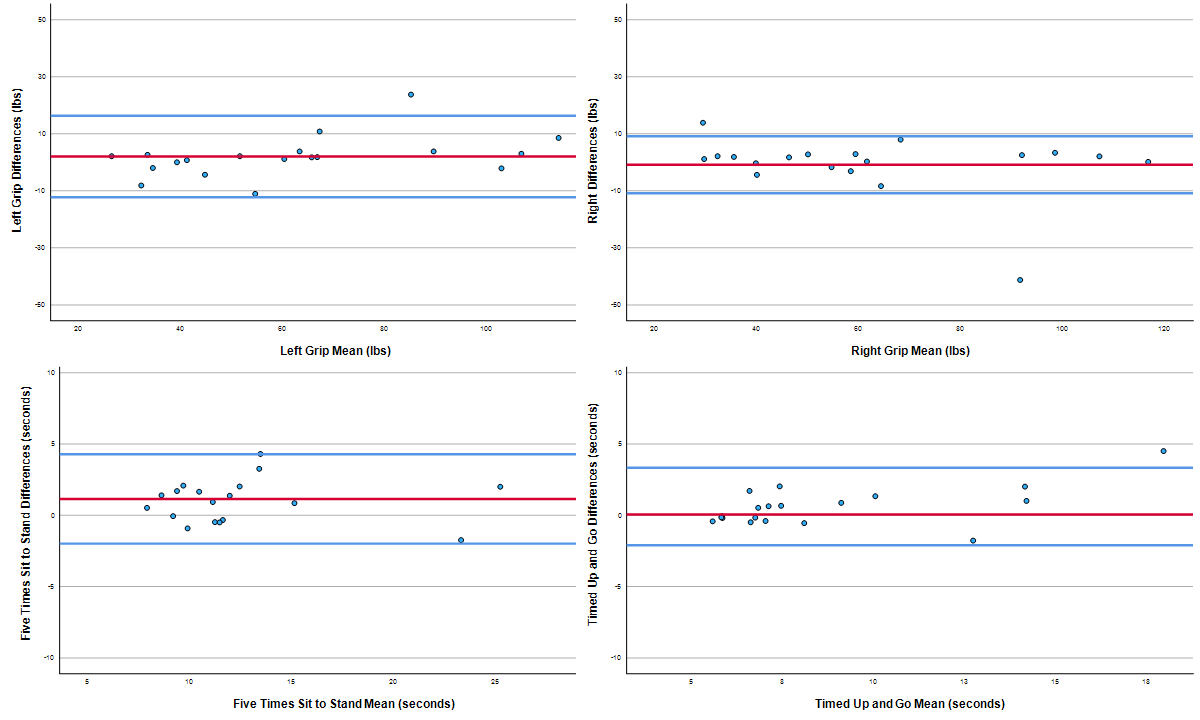
Bland-Altman plots for agreement between in-person and telehealth assessments of the hand grip strength test, the FTST, and the TUG test. FTST: five times sit-to-stand test; TUG: timed up-and-go.

Table 2 displays the ICC-v analysis results between in-person assessments and teleassessments for the hand grip strength test, the FTST, and the TUG test. Hand grip strength ICC_(2,3)_ analyses, with H_0_=0.75 (test value calculation vs a null hypothesis of good agreement), demonstrated statistically significant agreement between test conditions for both right-hand (ICC=0.96, 95% CI 0.9-0.99; *P*<.001) and left-hand (ICC=0.98, 95% CI 0.95-0.99; *P*<.001) grip strength. FTST test ICC_(2,1)_ analysis, with H_0_=0.75, demonstrated statistically significant agreement between test conditions (ICC=0.95, 95% CI=0.79-0.98; *P*=.01). However, the agreement result for the FTST was not statistically significant when tested against excellent agreement (*P*=.17). TUG ICC_(2,3)_ analysis, with H_0_=0.75, demonstrated statistically significant agreement between test conditions (ICC=0.92, 95% CI 0.79-0.98; *P*=.01). Agreement results remained statistically significant when tested against excellent agreement (H_0_=0.9). Bland-Altman plots (Figure 4) supported the ICC analyses and demonstrated strong agreement between conditions for hand grip strength, the FTST, and the TUG test.

**Table 2:** ICC-v^a^ for the hand grip strength test, the FTST^b^, and the TUG^c^ test.

Test	In-person assessment, mean (SD)	Teleassessment, mean (SD)	ICC-v (95% CI)	*P* value
Right-hand grip strength (lb)	63 (29.8)	61.9 (26.9)	0.96 (0.90-0.99)	<.001
Left-hand grip strength (lb)	61.8 (25.9)	64.2 (28.8)	0.98 (0.95-0.99)	<.001
FTST (seconds)	13.0 (5.9)	15.1 (7.7)	0.95 (0.79-0.98)	.01
TUG test (seconds)	8.5 (3.2)	9.2 (4.0)	0.92 (0.79-0.97)	.01

^a^ICC-v: intraclass correlation coefficient for validity.

^b^FTST: five times sit-to-stand test.

^c^TUG: timed up-and-go.

**Figure 5:**
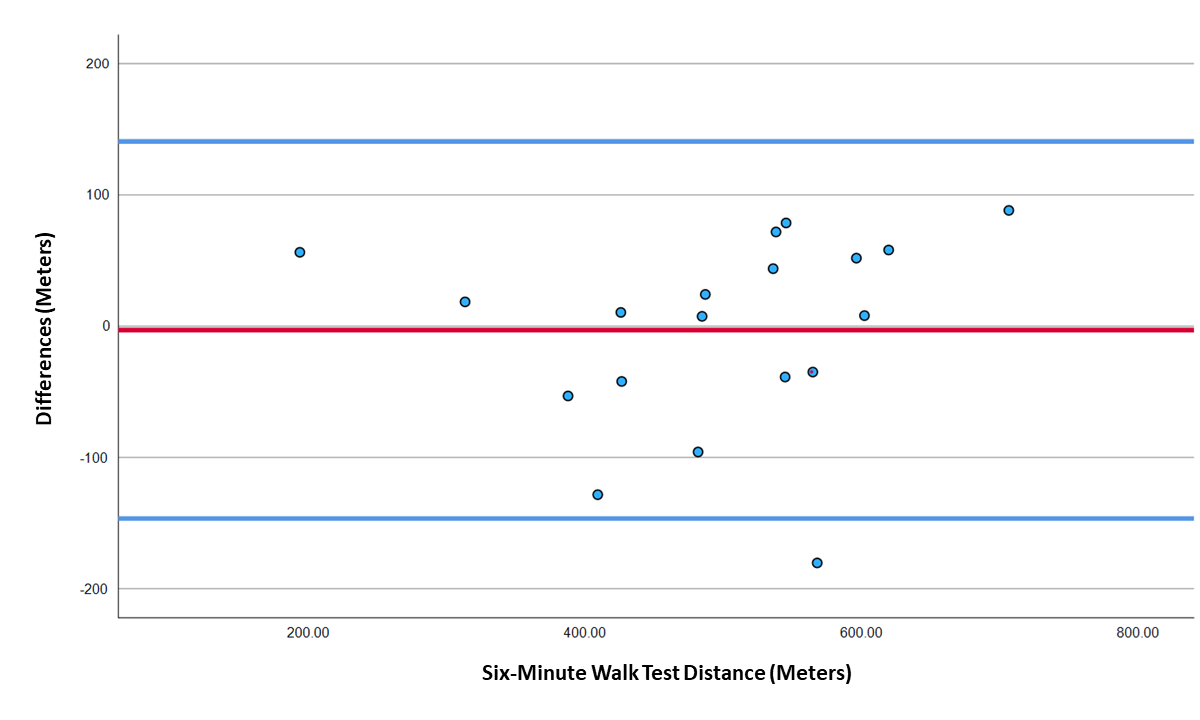
Bland-Altman plot for agreement in meters between the 6MWT and the converted HM6MWT with a 10.7 CF for laps to meters. 6MWT: 6-minute walk test; CF: conversion factor; HM6MWT: home-modified 6-minute walk test.

**Figure 6:**
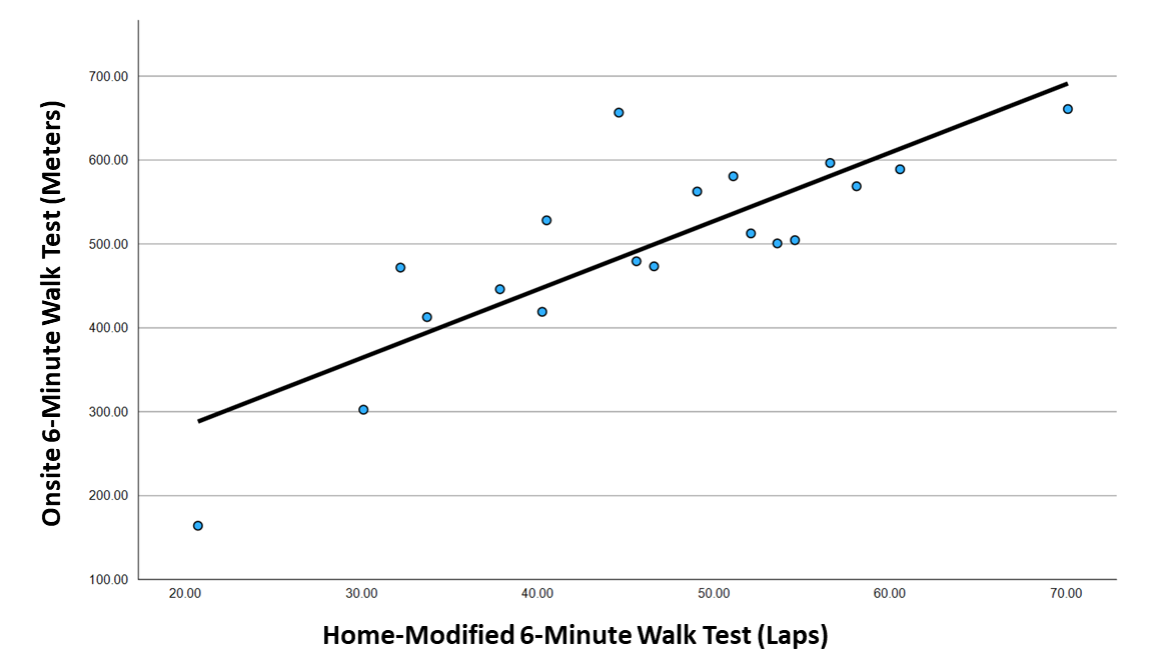
Linear regression analysis between the HM6MWT number of laps and the 6MWT in meters (r=0.825, 95% CI 0.593-0.930). The fitted line has a slope of 8.15 and a constant of 120.5. 6MWT: 6-minute walk test; HM6MWT: home-modified 6-minute walk test.

Table 3 displays the exploratory ICC-v analysis results between in-person assessments and teleassessments. Exploratory ICC_(2,1)_ analyses demonstrated that the conversion factor (CF) of a 10.7 lap multiplier provided the highest ICC agreement value (Table 3). However, the HM6MWT 10.7 lap multiplier ICC_(2,1)_, with H_0_=0.75, did not demonstrate statistically significant agreement with on-site 6MWT distances (*P*=.18). Teleassessment 10.7 ICC_(2,1)_ analysis, with H_0_=0.5 (fair agreement), showed a statistically significant agreement (ICC=0.83, 95% CI 0.62-0.93; *P*=.01). The Bland-Altman plot showed seemingly poor agreement for the teleassessment to either underestimate or overestimate walking distances compared to those obtained in person (Figure 5). Follow-up Pearson correlation analysis resulted in a strong positive correlation between both teleassessment laps counted (r=0.83, *P*<.001; Figure 6) and teleassessment walking distance with a 10.7 CF (r=0.83, *P*<.001) compared to on-site walking distances.

**Table 3:** ICC-v^a^ for the exploratory conversions of the HM6MWT^b^ and the 6MWT^c^.

Test	Converted distance (m), mean (SD)	6MWT distance (m), mean (SD)	ICC-v (95% CI)	*P* value
HM6MWT with x10.6 m/lap (m)	488 (128)	496 (119)	0.83 (0.62-0.93)	.18
HM6MWT with x10.7 m/lap (m)	493 (129)	496 (119)	0.83 (0.62-0.93)	.18
HM6MWT with x10.8 m/lap (m)	493 (131.8)	496 (119)	0.83 (0.61-0.93)	.18

^a^ICC-v: intraclass correlation coefficient for validity.

^b^HM6MWT: home-modified 6-minute walk test.

^c^6MWT: 6-minute walk test.

### Teleassessment Interrater Reliability and Disability (Purpose 2)

Hand grip strength ICC_(2,3)_ analyses, with H_0_=0.75 (good agreement), demonstrated statistically significant agreement between raters for both right-hand (ICC=1.0, 95% CI 1.0-1.0; *P*<.001) and left-hand (ICC=0.998, 95% CI 0.998-1; *P*<.001) grip strength. These results were the same when tested against excellent agreement (H_0_=0.9). For the rest of the teleassessment battery (FTST, TUG, and HM6MWT), the ICCs for reliability testing between the 2 raters (ICC-r) for the youth with CP are displayed in Table 4. The results demonstrated excellent agreement (tested against H_0_=0.9) for all 3 rater-dependent tests.

**Table 4:** ICC-r^a^ for the interrater reliability of the rater-dependent tests.

Test	Rater 1, mean (SD)	Rater 2, mean (SD)	ICC-r (95% CI)	*P* value
FTST^b^ (seconds)	17.0 (7.73)	16.9 (7.75)	0.998 (0.992-1.000)	<.001
TUG^c^ (seconds)	11.53 (4.57)	11.41 (4.65)	0.999 (0.997-1.000)	<.001
HM6MWT^d^ (laps)	36.85 (14)	36.75 (13.86)	0.999 (0.999-1.000)	<.001

^a^ICC-r: intraclass correlation coefficient for reliability.

^b^FTST: five times sit-to-stand test.

^c^TUG: timed up-and-go.

^d^HM6MWT: home-modified 6-minute walk test.

### Feasibility (Purpose 3)

For all participants, the time to complete the in-person battery (mean 16.9, SD 4.8 minutes) was on average 20% shorter (16.9/21.1 minutes) than the time to complete the teleassessment battery (mean 21.1, SD 5.9 minutes), and this difference was statistically significant (mean 4.16, SD 5.3 minutes; *P*=.003). Youth with CP took 45% longer (20.4/14.1 minutes) to complete the in-person assessments (mean 20.4, SD 2.4 minutes) than youth without disabilities (mean 14.1, SD 4.3 minutes), and this difference was statistically significant (mean difference 6.33, SD 3.8 minutes; *P*=.001). In addition, youth with CP took 33% longer (24.8/18.7 minutes) to complete the teleassessments (mean 24.8, SD 2.8 minutes) compared to youth without disabilities (mean 18.7, SD 5.7 minutes), with a mean difference of 6.11 (SD 5.4) minutes (*P*=.01). No adverse events, such as falls, occurred throughout the study.

Three participants with CP reported that the HM6MWT made them feel slightly dizzy and was more difficult because of the track’s limited length and the frequent turns resulting from it. Three participants without disabilities reported that the HM6MWT was more difficult due to the space limitation. This idea was supported by all 3 assessors, who observed that participants seemingly had to put more conscious effort into making the turns around the cones, particularly when walking at a fast speed. The assessors also noted that cognitive disability seemed to cause variability in turns. The 1 (5%) participant with mild-to-moderate cognitive disability walked in different paths around the cones on each lap: some big paths around the cones and some small tight paths. Some participants adopted head-and-eye-focusing strategies to prevent feeling nauseated when turning. Participants generally reported that the tests were similar between the 2 settings, except for the HM6MWT.

## Discussion

### Principal Findings

This study investigated the feasibility, validity, and reliability of an inclusive telehealth battery of physical fitness and function among a cohort of youth with and without disabilities. A strength of the teleassessment battery was that it could be delivered with minimal, low-cost supplies. The battery included 4 web-modified tests, and the results of these tests were compared with how they were typically conducted on-site at a laboratory. All 4 web-based tests were modified so that they could be delivered through videoconferencing and within a small home environment. Most modifications were minor, except for the HM6MWT, which included the largest modification: a long-distance track that was converted to a small straight-path walkway. Overall, study findings suggested that the teleassessment battery had accessible feasibility, as indicated by safety and convenience. The mean time for completing the assessments was short, under 30 minutes. No falls, problems, or other adverse events occurred. Findings warrant a true examination of feasibility in a less controlled environment: the participants’ homes. Of note, the study findings showed that a novice and an expert assessor can achieve similar results when conducting the web-based assessments (excellent interrater reliability), which has important practical implications for implementation. First, highly experienced personnel may not be necessary to conduct the teleassessments. Second, a participant who completes an intervention does not need to be scored by the same rater who scored their baseline assessments, thereby reducing scheduling constraints and the burden on research staff. Most importantly, findings largely demonstrated good-to-excellent convergent validity between the tele- and in-person assessments.

### Comparison With Previous Work

Regarding validity, the web-modified versions for the hand grip strength test, the FTST, and the TUG test had excellent agreement with scores obtained from the in-person assessments. Researchers and health professionals may feel confident in performing these tests through videoconferencing, when the participant’s environment conforms with the study procedures. As for the HM6MWT, the findings are less clear. The HM6MWT demonstrated only fair absolute agreement with in-person assessments, and this was when analyzed with the best-possible CF for transforming laps walked into walking distance in meters. Bland-Altman plots showed that the web-modified test overestimated or underestimated walking distances by greater than 100 m, which is substantially large, given that the mean walking distance for this age group is 496 m. This finding indicated that the HM6MWT distance in meters (converted from laps) should not be compared with the distance in meters obtained from an in-person 6MWT. Nevertheless, correlation analysis demonstrated strong agreement between the 2 types of test conditions, indicating that the web-modified 6MWT could still be considered a valid assessment. Consequently, the HM6MWT could still potentially be useful for measuring pre- and postintervention changes in walking performance. We would recommend that health professionals consider the number of laps counted as the outcome measure, as opposed to the walking distance obtained through a CF, to avoid confusion with interpretation of these results with in-person walking tests. Of course, further research is needed to support the validity of the HM6MWT. For example, given that the 6MWT is often used as an indirect indicator of cardiorespiratory endurance in clinical populations, there is a need to explore whether changes in HM6MWT laps over time are comparable with changes in cardiorespiratory fitness (criterion validity). There is a dire need for home-based assessments for cardiorespiratory fitness, given that there are (to the best of our knowledge) no scientifically sound assessments for measuring cardiorespiratory fitness remotely at home without specialized equipment and personnel.

Study findings are comparable with those among different age groups. One study reported that a videoconference assessment of the FTST is extremely reliable (ICC>0.9) and the TUG test is highly reliable (ICC>0.7) among older adults [13]. Another study among adults (mean age 37, SD 12.5 years) demonstrated excellent agreement for grip strength (ICC 0.99, 95% CI 0.99-0.99), good agreement for the FTST (ICC 0.84, 95% CI 0.75-0.9), and fair agreement for the TUG test (ICC 0.64, 95% CI 0.47-0.77). The study concluded that untimed measures, such as grip strength, have excellent reliability. For the timed outcome measures, comparison of in-person and telehealth outcomes was not recommended [19]. Likewise, study findings for interrater reliability are consistent with those reported by other investigations that included older adults without disabilities [17,18]. Regarding modifications to the conventional 6MWT, a previous study had children with CP perform the 6MWT over 15 and 30 m courses [67]. The authors concluded that a shorter and narrower walking course could result in more turning and less straight walking paths, both of which could negatively affect or add volatility to the walking distances [67]. This could explain the variable differences observed between the HM6MWT and 6MWT distance results in our study.

### Future Considerations

It is important to note that not all youth will prefer teleassessments versus in-person assessments. We would recommend that future trials include both options for youth to complete the assessments. Moreover, our study included simple assessments with minimal verbal instructions. Many exercise assessments require specialized equipment and instructions and complex movements, which will make these assessments difficult to perform via videoconferencing. There is a need to identify innovative measurement methods or technology that can address logistical issues for more complex tasks.

### Limitations and Future Directions

This exploratory pilot study had inherent limitations. First, the sample size, although statistically powered for the primary analyses, was clearly not large enough to be a truly representative sample. One of the most notable limitations of our study is that the 9 youth with disabilities all had CP as their preexisting condition and were ambulatory. The result of only youth with CP was a coincidence. Although CP is an umbrella term with overlapping neuromuscular characteristics with traumatic brain injury, spinal cord injury, or other neuromuscular diseases, diversifying the study population would further promote the adoption of teleassessment as a modality of research and clinical assessment. Future research is also needed to identify home-based measures of physical fitness and function for people who are nonambulatory. People who are nonambulatory are underrepresented in exercise trials among people with disabilities and are often excluded from participation [1]. Of note, the study sample was also highly literate with videoconferencing, which will likely not be generalizable to the population.

Second, the study was not conducted in a real-world setting. The teleassessments were conducted in a controlled setting within a research laboratory where Wi-Fi and equipment were well maintained and set up by laboratory staff for use. The necessary space for the teleassessments (approx. 10 × 15 square feet) may also not be available without obstacles in a person’s home. Thus, study findings for feasibility will likely not represent the technical challenges that people may encounter outside the research environment—for example, shipping the equipment to the participants’ homes and calibrating equipment.

Third, this study focused only on convergent validity and interrater reliability. Other aspects of psychometric properties, such as responsiveness and the level of measurements, should be investigated, ideally with clinical populations with disabilities in their youth.

Finally, although the order of the test conditions was randomized, since all tests were performed in a single session, there is still the possibility that a learning effect influenced the results.

### Conclusion

This study demonstrated that a teleassessment battery is feasible and certain components of it may be suitable for measuring fitness and function among ambulatory youth with CP and without disabilities. Convergent validity was excellent for the hand grip strength test and good for the FTST and the TUG test. The HM6WT requires further investigation or supportive measures prior to being used in a clinical trial. Standardized instructions for conducting the teleassessments are included in Multimedia Appendix 1. This study fills a gap in research on the youth age group, who are often neglected in research due to their presumed healthiness, not belonging to either children or adults in the narrow sense.

## Appendix

Multimedia Appendix 1 Manual of procedures.

## Abbreviations

6MWT: 6-minute walk test
CF: conversion factor
CP: cerebral palsy
FTST: five times sit-to-stand test
HM6MWT: home-modified 6-minute walk test
ICC: intraclass correlation coefficient
ICC-r: intraclass correlation coefficient for reliability
ICC-v: intraclass correlation coefficient for validity
TUG: timed up-and-go

## Data Availability

The data sets generated and analyzed during this study are not publicly available due but are available from the corresponding author upon reasonable request.
