# Supplementary material for: Validity and Reliability of a Telehealth Physical Fitness and Functional Assessment Battery for Ambulatory Youth With and Without Mobility Disabilities: Observational Measurement Study
Source: JMIR Rehabil Assist Technol. 2024 Feb 12;11:e50582. doi: 10.2196/50582 (PMC10897795; doi:10.2196/50582)
Supplement: Multimedia Appendix 1 [file rehab_v11i1e50582_app1.docx]

***Setup***

- Turn on laptop and place in teleassessment room (minimum of 10 x 15 feet of space, to resemble a modest estimate of an average living room)
- Create a Zoom meeting and have the laptop join the meeting, use another computer/device to talk to the laptop (assessor side) **MAKE SURE THE MEETING IS RECORDED**
- Ensure teleassessment room has measuring tape (rolled up) and mini-disc cone available on the table with laptop
- Ensure participant has the hand grip dynamometer (you can use the same one for both rooms)
- Ensure person uses chair without arms for the Timed Up and Go and the Five Times Sit to Stand


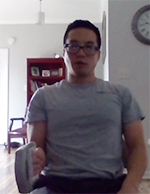
***Grip Strength (90 °) Test***

Equipment: a chair with arm, a digital hand dynamometer

Camera view includes the front view of participant’s upper body

Have the participant sit in a stationary chair or a wheelchair and use a hand dynamometer from the teleassesssment equipment package

Have participant adjust grip so that the pull bar is under the intermediate phalanges

Set handgrip to lbs


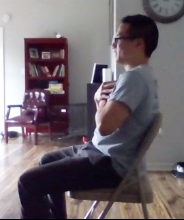
 Ensure the screen of device is turned away from the individual's view (face outward)

For each trial say, “Okay, on the count of 3 I want you to squeeze as hard as you possibly can. After you squeeze, please show me the screen of the device so I can record the number. Are you ready? Okay, 3, 2, 1, Squeeze.”

Have participant show force in lbs immediately after each trial

***Five Times Sit to Stand Test***

Equipment: a chair (you may use a chair with arms for safety but discourage participant from using if unnecessary)


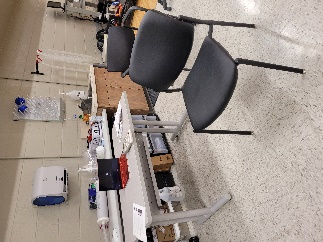
Camera view includes the side view of participant’s entire body (at least, shoulders, hip, and knees)

Ensure feet are touching the floor

Have the participant sit in a stationary chair or a wheelchair

Participant needs to get back to neutral at 90 degrees with the body, for it to count

***Timed Up & Go Test***

Equipment: a chair with arm, a mini disc cone, a 118-inch soft measuring tape

- - 1. Camera view of back of the participant (laptop on table behind chair)
    2.
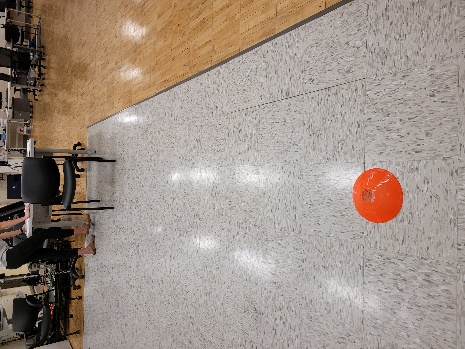
**Ensure at least 3 feet from the laptop/table to the persons feet, to allow adequate space for walking and turning around while performing the 6MWT (same setup)**
    3. Have the participant lay down the measuring tape starting at the tip of their toes when sitting in a chair, then place the cone at the end of the measuring tape. Then, remove the measuring tape and sit down in the chair. ***Ideally, the measuring tape should have minimal twists/wrinkles.***
    4.
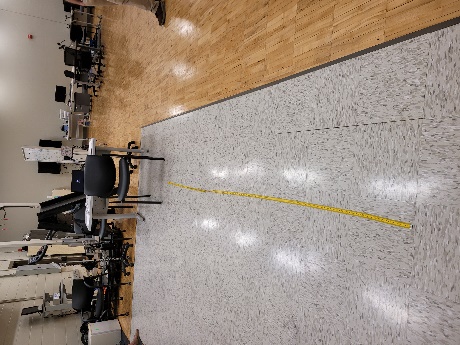
Participant instructions: “Okay, for this timed up and go test, I want you to stand up and walk at your maximal comfortable speed around the cone, back to the chair, and sit down. I will time how long it takes to complete the task. Please remember to be safe during the test, especially when turning. Are you ready? Okay, 3,2,1, start.”

***Six-Minute Walk Test (6MWT)***

***Tele-assessment***

Equipment: two cones and a 118-inch adhesive measuring ruler

- - 1. ***Camera view is the same as in the TUG test.***
    2. *Have the participant place one cone at their feet while sitting in the chair (the starting cone)*
    3. *Have the participant remove the chair (push the chair to the side)*
    4. *Inform participant that there should be no more than one twist in the tape, have them lay out the tape again if necessary. If balance is an issue, they may require a caregiver to step on one end of the tape to hold it down while they or the caregiver lay it out.*
    5. **INSTRUCTIONS:** “For this test, the objective is to walk AS FAR AS POSSIBLE for 6 minutes. You may slow down if necessary. If you stop, please continue walking again as soon as possible. I will keep you informed of the time. When I say stop, please stop and stand still so that I can see where you ended. Are you ready? Okay, 3,2,1, start.”
    6. Time 6 minutes with your stopwatch
    7. Count laps on the data collection form or a piece of paper
    8. Guess the fraction of the last lap (e.g., half a lap = 0.5)

***In-Person***

- - 1. *Have participant start at one end of the track*
    2. *Have a research assistant walk behind the participant with a trundle wheel*
    3. **INSTRUCTIONS:** “For this test, the objective is to walk AS FAR AS POSSIBLE for 6 minutes. You may slow down if necessary. If you stop, please continue walking again as soon as possible. I will keep you informed of the time. When I say stop, please stop and stand still so that I can see where you ended. Are you ready? Okay, 3,2,1, start.”
    4. Give verbal notices of the time at minute 3, 4 and 5
    5. Time 6 minutes with your stopwatch
